# Chondrosarcoma mimicking an adnexal mass: A very rare case report

**DOI:** 10.4274/tjod.27096

**Published:** 2017-03-15

**Authors:** Hüseyin Çağlayan Özcan, Aynur Mustafa, Zehra Bozdağ, Seyhun Sucu, Özcan Balat

**Affiliations:** 1 Gaziantep University Faculty of Medicine, Department of Obstetrics and Gynecology, Gaziantep, Turkey; 2 Gaziantep University Faculty of Medicine, Department of Pathology, Gaziantep, Turkey

**Keywords:** Chondrosarcoma, diagnostic errors, ovarian mass

## Abstract

Chondrosarcoma is considered as a common primary bone sarcoma. These sarcomas can form large masses without any specific symptoms because there are no barriers in pelvic anatomy to prevent the enlargement of tumors, and can mimic ovarian masses. We present a pelvic chondrosarcoma in a woman aged 37 years who was misdiagnosed as having an ovarian mass due to the limited information obtained from imaging studies. Pelvic chondrosarcoma should be considered in patients who have pelvic masses with solid components. It should be kept in mind that interventions should be performed at centers where there are orthopedic surgeons with experience of this subject.

## INTRODUCTION

Chondrosarcoma is considered as a common primary bone sarcoma, which is ranked as the third primary bone malignancy. One in five cases of bone sarcomas are due to chondrosarcoma, and half of all cases affect the pelvis^(1)^. Moreover, this tumor arises predominantly after the second decade with a peak in the middle-aged period. Many heterogeneous groups of neoplasms are related to chondrosarcoma and cartilage matrix production is a typical feature of this tumor^(2)^. These sarcomas form large masses without any specific symptoms because there are no barriers in the pelvic anatomy to prevent the enlargement of tumors^(3)^. In this case report, we present a patient who had a tumor of undefined origin according to preoperative imaging methods, and a pelvic chondrosarcoma mimicking an adnexal mass. To the best of our knowledge, this is only the third case of pelvic chondrosarcoma in the English literature of obstetrics and gynecology.

## CASE REPORT

**Figure 1:**
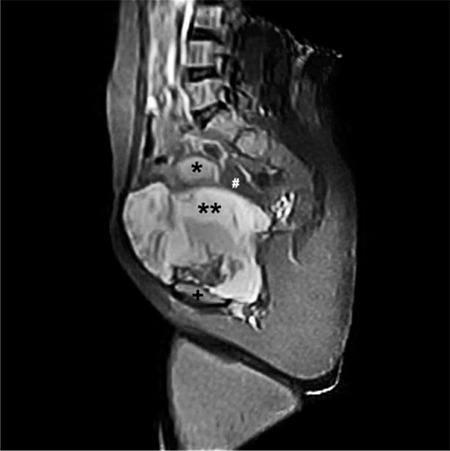
Magnetic resonance imaging showing a solid mass with 20x10 cm in diameter including calcified areas, and a mass consistent with myoma of 3.5 cm in diameter was observed in the cervico-isthmic region of the uterus

**Figure 2:**
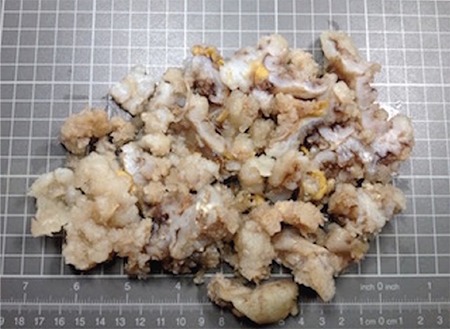
The macroscopic appearance of the lesion, which is seen in pieces, and has the solid-brilliant cartilaginous appearance of cross-sectional surface

**Figure 3:**
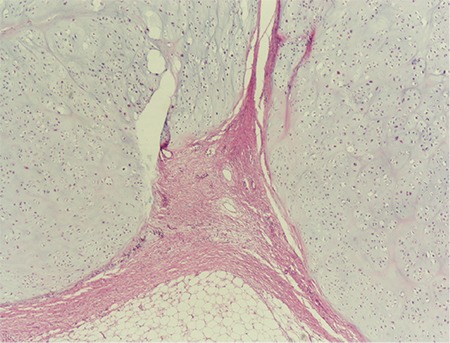
Microscopic appearance of the lesion, which was separated by fibrous septa, and was formed by nodular infiltrating, atypical chondrocytes, hematoxylin and eosin x40

A 37-year-old single woman presented to our clinic with symptoms of pelvic pain, abdominal distension, and vaginal bleeding. During the pelvic examination, a mass that filled the pelvis was detected. The carcinoma antigen 125 (CA125) level was 40 U/mL. Ultrasonographic and magnetic resonance imaging (MRI) examinations revealed a solid mass 20x10 cm in diameter including calcified areas, and a mass consistent with myoma of 3.5 cm in diameter was observed in the cervico-isthmic region of the uterus. The endometrial thickness was 8 mm (Figure 1). During surgery, a mass of 18x8 cm was observed that was displacing the uterus and extending from the symphysis pubis to the pelvis with a myoma of 3.5 cm in diameter, posterior to the uterus corpus. The ovaries were normal on inspection. Specialists from the urology and orthopedics departments were consulted intraoperatively and it was decided that the tumor originated from the pelvic bone. Partial resections with myomectomy were performed because the tumor could not be completely removed (Figure 2). The postoperative pathologic examination was reported as grade 1, well-differentiated chondrosarcoma (Figure 3). The orthopedics department requested computerized tomography (CT) imaging, which revealed masses consistent with metastasis in the liver, and a 9x8 cm diameter mass in the pubic area. The patient was referred to another healthcare unit because a skilled orthopedics team with experience of pelvic chondrosarcoma was unavailable.

## DISCUSSION

That a pelvic chondrosarcoma could masquerade as an ovarian mass was considered the peculiar part of our case. This camouflage exposes the limited role of imaging techniques and tumor markers in diagnosing pelvic tumors. Generally, chondrosarcoma has a silent course due to the special pelvic structure, which allows masses to grow feasibly without any boundaries and only become symptomatic after enlarging enough, as in our case. Thus, this tumor has a larger size in comparison with other pelvic masses when the diagnosis was established; the mean size is commonly 11 cm^(4)^. After reviewing all operated cases of pelvic mass in our department within the last 15 years, chondrosarcoma was the only case that was misdiagnosed as an ovarian mass, which reflects the sporadic incidence of this condition. Although all available imaging techniques, even CT and MRI, were used to assist us in mapping this tumor, we could not identify the exact margins, neither the exact organ from which this tumor derived. Moreover, the elevated CA125 was another misleading factor that increased our suspicion of ovarian mass. The surgical management of chondrosarcoma is a destructive operation for orthopedic surgeons due to the following principles: increased risk of vital organ injuries, high susceptibility to damage pelvic structural stability, challenging anatomic interactions of the pelvis, and devastating extension of the tumor. The mass could not be completely removed and the patient was considered as non-resectable and referred to another orthopedics clinic because we did not have an orthopedic surgical team skilled in pelvic chondrosarcoma. Vast majority of chondrosarcoma become symptomatic after reaching a large size and this can be explained by the slow growth rate of this these tumors^(5)^. Grade 1 chondrosarcomas consist of profuse hyaline cartilage matrix surrounding a little cellular mass and metastasize infrequently^(1)^. In our case, the pathologic result was grade 1 chondrosarcoma, and there were metastatic lesions observed in the liver in abdominal tomography. Pelvic chondrosarcomas can appear in various pathologic neoplasms, other than pure chondrosarcoma arising from the pelvic bone, such as chondrogenic tumors of the ovaries or heterologous carcinosarcomas of the uterus. However, all mentioned types have very low incidence rates^(6)^. Moreover, mixed mesodermal tumors, commonly called carcinosarcoma, another manifestation of chondrosarcoma, are the most common heterologous sarcomas, originating from the uterus in most cases. The histologic appearance is a mixture of ectoderm and mesoderm-derived tissues. Either homologous or heterologous mesodermal tissue can be commonly found to be high grade. Heterologous tumors consist of a differentiated mesenchymal component accompanied by endometrial, stromal or undifferentiated sarcomas. Occasionally, these uterine tumors may enlarge and convert to giant pelvic masses that can destroy the uterine structure to the point where it can become unrecognizable^(7)^. The cornerstone of treatment in managing this tumor is wide surgical excision, that still first line treatment^(8)^. After performing extensive intralesional curettage, local adjuvant treatment has encouraging long-term outcomes and adequate control of local recurrence in low-grade chondrosarcomas. Local adjuvant treatment is only effective in cases with well-defined boundaries with no extension beyond the bone^(9)^. However, giant tumor or pelvic localization of chondrosarcoma can alter the treatment method, even in low-grade conditions, as in our case, which require wide resection as a first-line management, rather than intralesional curettage^(1)^.

## CONCLUSION

Pelvic chondrosarcoma should be considered in patients who have pelvic masses with solid components because preoperative evaluations (imaging studies, pelvic examination) in daily practice may be inadequate for the diagnosis of pelvic masses. As a consequence, it should be kept in mind that interventions should be performed at centers where there are orthopedic surgeons with experience of this subject.
